# Evaluation of PD-L1 and tumor infiltrating lymphocytes in paired pretreatment biopsies and post neoadjuvant chemotherapy surgical specimens of breast carcinoma

**DOI:** 10.1038/s41598-021-00944-w

**Published:** 2021-11-18

**Authors:** Lucas Grecco Hoffmann, Luis Otavio Sarian, José Vassallo, Geisilene Russano de Paiva Silva, Susana Oliveira Botelho Ramalho, Amanda Canato Ferracini, Karina da Silva Araujo, Rodrigo Menezes Jales, Deayra Emyle Figueira, Sophie Derchain

**Affiliations:** 1 Postgraduate Program in Tocogynecology, Faculty of Medical Sciences, State University of Campinas (UNICAMP), Campinas, 13083-887 Brazil; 2 Department of Obstetrics and Gynecology, Faculty of Medical Sciences, Women’s Hospital Prof Dr José Aristodemo Pinotti (CAISM), State University of Campinas (UNICAMP), Campinas, 13083-970 Brazil; 3 Laboratory of Investigative Pathology, CIPED, Faculty of Medical Sciences, State University of Campinas (UNICAMP), Campinas, 13083-887 Brazil; 4 Laboratory of Molecular and Investigative Pathology – LAPE, Faculty of Medical Sciences, State University of Campinas (UNICAMP), Campinas, 13083-970 Brazil; 5 Department of Oncology, Woman’s Hospital Prof Dr José Aristodemo Pinotti (CAISM), Faculty of Medical Sciences, State University of Campinas (UNICAMP), Campinas, 13083-970 Brazil; 6 Multipat Anatomic Pathology Laboratory, Campinas, 13086-130 Brazil

**Keywords:** Breast cancer, Predictive markers, Prognostic markers, Tumour immunology, Immunosurveillance, Cancer microenvironment, Cancer, Breast cancer

## Abstract

Herein it was evaluated the impact of PD-L1 immunohistochemical expression and stromal tumor-infiltrating lymphocyte (sTIL) counts in pretreatment needle core biopsy on response to neoadjuvant chemotherapy (NACT) for patients with breast carcinomas (BC). In 127 paired pre- and post-NACT BC specimens, immunohistochemical expression of PD-L1 was evaluated in stroma and in neoplastic cells. In the same samples sTILs were semi-quantified in tumor stroma. Post-NACT specimens were histologically rated as having residual cancer burden (RCB of any degree), or with complete pathological response (pCR). PD-L1 expression and higher sTIL counts were associated with histological grade 3 BC. PD-L1 expression was also associated with the non-luminal-HER2+ and triple negative immunohistochemical profiles of BC. Pathological complete response was associated with histological grade 3 tumors, and with the non-luminal-HER2+ and triple negative profiles. Additionally, our results support an association between PD-L1 expression and pCR to NACT. It was also observed that there is a trend to reduction of sTIL counts in the post-NACT specimens of patients with pCR. Of note, PD-L1 was expressed in half of the hormone receptor positive cases, a finding that might expand the potential use of immune checkpoint inhibitors for BC patients.

## Introduction

Breast cancer is the most common malignancy among women worldwide, excluding non-melanoma skin cancer. This neoplasia accounts for about a third of all cancers and represents the first most frequent cause of death^1^. In Brazil, breast cancer has an estimated incidence of 66,280 cases for 2020, and a mortality rate of 17,572 patients in 2018^2^. Treatment of breast cancer includes surgery, radiation therapy, chemotherapy, hormone therapy and targeted therapy^3–5^. Breast cancer is a heterogeneous disease classified into different molecular subtypes^6,7^. An immunohistochemical surrogate panel was proposed to guarantee a wider clinical usage of the molecular typing. This panel includes markers for estrogen (ER) and progesterone receptors (PR), the cell proliferation associated antigen Ki67 and the epidermal growth factor receptor c-erb-B2/Her-2-neu (HER2). A dubious immunohistochemical expression of HER2 implies investigation of gene amplification by fluorescence in situ hybridization^8^.

Although the classification of breast carcinomas into molecular or immunohistochemical subtypes has contributed to a better stratification of patients for different therapeutic regimens^9^, a significant number of patients do not benefit from chemotherapy^10^. The major obstacle in treating any cancer with chemotherapy is the development of chemoresistance, leading to disease progression and metastasis^5,11–15^. Drug resistance is perhaps the main cause of death associated with cancer and brings significant harm to therapeutic interventions.

This drawback has led to the development of novel therapeutic choices, among which immunotherapy involving the PD-1/PD-L1 axis blockade. Further, PD-L1 expression in breast carcinomas, associated with increased density of stroma tumor-infiltrating lymphocytes (sTILs), shows a plausible predictive value for response to chemotherapy, especially in the context of neoadjuvance^16–18^.

Previous studies have shown expression of PD-L1 in both tumor epithelial cells and sTILs. However, there is still much controversy in defining which pattern of PD-L1 expression is clinically relevant. In positive cases, an irregular distribution pattern, limited to small aggregates of tumoral and/or immune cells predominates^19,20^. Another challenging issue related to the immunohistochemical evaluation of PD-L1 is the antibody clone and the automated platform in which the reaction is performed. Multiple clones and platforms have been used for different tumor types, as SP-142 and SP-263 (Roche), 22C3 and 28-8 (Dako), ZR3 (Cell Marque), QR1 (Quartett) and others^21,22^. Finally, subjectivity in scoring the reaction has been verified among pathologists^23^.

In this scenario, studies in different settings are needed to investigate the clinical value of PD-L1 expression and sTIL counts in breast cancer. The present study was designed to evaluate the relationship between PD-L1 expression and sTIL counts before and after neoadjuvant chemotherapy in women with breast cancer, and their relationship with pathological response.

## Materials and methods

### Study design, setting and subjects

The present cohort study was conducted at the Women’s Hospital (Hospital da Mulher Professor José Aristodemo Pinotti, Centro de Atenção Integral à Saúde da Mulher—CAISM), State University of Campinas (UNICAMP), São Paulo, Brazil. Women with invasive breast carcinoma who underwent neoadjuvant chemotherapy (NACT) and post-NACT surgery between June 2016 and June 2019 were included. None of them received endocrine therapy in the neoadjuvant scenario. The study was approved by the Research Ethics Committee of Campinas State University (CAAE: 85013718.1.0000.5404). All procedures were carried out according to the Helsinki Declaration and its later amendments. Biological samples were stored at the Biobank number 56 from CAISM. All women signed the informed consent before having their biological samples deposited in the institutional biobank. The diagnosis of breast carcinoma was performed on specimens obtained by percutaneous needle (core) biopsy under ultrasound view. Criteria for indicating NACT included histological subtype, stage and age. NACT followed the institutional clinical protocol, considering the immunohistochemistry based molecular subtype of breast cancer.

The following regimens are indicated as NACT at our hospital: for luminal BC, four cycles of standard chemotherapy with usual anthracycline plus cyclophosphamide, with 21 days intervals, followed by weekly dose of taxane for 12 weeks, the (ACT) regimen^24^. For HER2 positive BC, the same ACT regimen is applied, plus four cycles of trastuzumab every 21 days, during NACT; then, trastuzumab is given as adjuvant therapy to complete 17 cycles^25^. Carboplatin is added to standard NACT for triple negative breast cancer (TNBC), to improve pathological complete response (pCR), although this indication is controversial in the literature^26^. After NACT all women underwent surgical treatment (mastectomy or quadrantectomy with sentinel lymph node biopsy or axillary lymph node dissection), according to the standard institutional protocol. Patients who abandoned treatment and those without available tumor tissue on pre-treatment archival specimens were excluded.

Among 127 women included in the study, three presented bilateral carcinoma. Clinicopathological data, as age, ethnicity, menopausal status, pregnancy, lactation, hormone replacement, family background of breast or ovarian cancer, smoking, clinical tumor stage, and clinical axillary lymph node status were obtained at medical consultation, registered in the medical records and listed on a data collection form. Clinical staging was performed according to the International Federation of Gynecology and Obstetrics system^27^. Response to chemotherapy was assessed in the surgical specimen post-NACT, according to the Residual Cancer Burden (RCB) online calculator. This reproducible and clinically validated approach allowed scoring of response to therapy as: complete pathological response (pCR), RCB-I (minimum residual disease), RCB-II (moderate residual disease), and RCB-III (extensive residual disease). The pathological reports provided the final residual tumor dimensions (in mm), the percentage of cancer cell areas in the residual tumor bed, and the proportion of in situ component. In addition, the number of positive lymph nodes and the diameter (mm) of the largest nodal metastasis had been informed^28^. For statistical analyses, cases were clustered into: (a) cases with complete pathological response (pCR), and (b) cases with residual breast carcinoma (RCB-I to RCB-III).

### Histopathology and immunohistochemical evaluation

Tissue samples were collected either by percutaneous needle core biopsy (pre-treatment specimens), or during surgical resection after NACT (post-treatment specimens). All samples were fixed in 10% buffered formalin and paraffin-embedded. Hematoxylin–eosin-stained sections were reviewed to confirm the histological diagnosis in the core needle biopsy and in post-NACT tissues. In every case, histological diagnoses were established according to the World Health Organization (WHO) criteria^29^. Histological non special and special types were included; tumor grade and the presence of vascular invasion were revised.

Stromal tumor-infiltrating lymphocytes (sTILs) were assessed on hematoxylin–eosin-stained slides from matched pre-treatment core needle biopsies and post-neoadjuvant chemotherapy resection specimens. sTILs were assessed according to the guidelines of the Immuno-Oncology Biomarker Working Group^29^.Although the guidelines for sTILs evaluation have been established for initial diagnostic specimens of tumors, they were also used for residual disease. Stromal TILs were defined as the percentage of tumor stroma area occupied by mononuclear inflammatory cells in the tissue samples. After NACT, in women with residual cancer, sTILs were evaluated in the residual tumor bed, defined as the largest cross-sectional area of residual invasive tumor cells^30^. In women with pCR, sTILs were assessed in the tumor bed. The criteria described by Dieci et al.^30^ were used to group sTILs intensity: absent, mild (> 1% to 10%), moderate (11% to 60%), and intense (> 60%). Only sTILs in association to invasive tumors were considered; areas of ductal carcinoma in situ and normal breast lobules were not addressed for sTILs scoring^31^.

Conventional manual immunohistochemical technique was performed, as follows: tissue sections were deparaffinized in xylene and rehydrated in alcohol gradient; antigen retrieval was made in citrate buffer (pH 6.0) in a regular microwave at 95º C for 30 min; endogenous peroxidase activity was blocked with 0.3% hydrogen peroxidase; then, slides were incubated overnight with the primary antibodies at 8° C; the next morning, primary antibodies were rinsed away with phosphate buffer saline, and the biotin-free peroxidase-conjugated polymer system was applied for one hour at room temperature; freshly prepared 3–3′-diaminobenzidine tetrahydrochloride was used to reveal peroxidase bound to immunocomplexes for 25 min; counterstain was achieved using Harris hematoxylin, followed by dehydration, clearing, and mounting. Positive and negative controls were run in each batch.

Primary antibodies used for the evaluation of molecular types of breast cancer were: anti-estrogen receptor (ER, clone 1D5, diluted at 1:1000), anti-progesterone receptor (PR, clone PR636, diluted at 1:800), anti-HER2 (polyclonal, code A0485, diluted at 1:1100), and proliferation marker Ki67 (clone MIB1, diluted at 1:500). All antibodies and the detection system Envision Flex were provided by Dako—Agilent, Santa Clara, CA, USA. For the evaluation of PD-L1 expression, an anti-PD-L1 rabbit monoclonal antibody was used (clone ZR3; diluted at 1:75, Cell Marque – Sigma-Aldrich, MO, USA)^32^; in this case, antigen retrieval was made using Tris–EDTA buffer, pH 9.0.

Evaluation of ER and PR was performed according to Allison et al.^33^, on the diagnostic (pre-treatment) specimens. Cases were considered positive if 1% or more tumor cells were positive. For Ki67, results were represented by the percentage of positive tumor cells in hot spots^34^. Human epidermal growth factor receptor 2 (HER2) staining was scored as 0 + /1 + (negative), 2 + (equivocal), or 3 + (positive). Equivocal (2 +) cases were further submitted to fluorescence in situ hybridization (FISH) to detect gene amplification in a reference Laboratory, according to the recommendations of the American Society of Clinical Oncology/College of American Pathologists (ASCO/CAP)^35^. To address tumor heterogeneity, in cases negative for hormone receptors and/or HER2 in immunohistochemistry performed on the pre-treatment tissue sample, immunohistochemistry was remade on the surgical specimens with residual disease for subtype confirmation^36^.

Cases were grouped according to the reactivity with these markers in four groups: *luminal-like* (hormone receptor positive/HER2 negative); *luminal-HER2* (hormone receptor positive/HER2 positive); *non-luminal-HER2* (hormone receptors negative/HER2 positive); *triple negative* (hormone receptors negative/HER2 negative)^8^.

PD-L1 assessment was visually estimated on both pre- and post-NACT tissue specimens. Membranous and cytoplasmic staining of epithelial tumor cells and stroma cells were evaluated. A case was considered positive if more than 1% of positive cells were present, in one or both components^37,38^. In the presence of residual tumor, comparison of PD-L1 expression between pre- and post-NACT specimens was made in tumor and stromal cells. In patients with pCR, only stromal components were compared between pre and post NACT specimens.

As evaluation of PD-L1 immunostaining is not part of the pathologist’s customary routine, it was independently carried out by two pathologists for all 127 matched specimens (LH and KA). After a period of co-observation training, both pathologists estimated the expression of this marker individually. Then, the interobserver agreement was calculated. For discordant cases, a consensus of both pathologists was reached at the coobservation microscope. The same routine was adopted for post-NACT specimens. All other microscopical evaluations were made as standardized in pathology practice.

### Statistical analysis

Data were analyzed using the R Environment for Statistical Computing Software^39^. Significance was set at *p* < 0.05, with 95% confidence intervals (CIs). First, we assessed the relationship between key clinical and pathological features and PD-L1, sTILs, pathological response status, using a bivariate binomial model; odds ratios and p-values for the associations were calculated. Next, we compared the pre-treatment status (biopsy fragment) and post-neoadjuvant chemotherapy, paired with the sTIL counts (evaluating in both cases with residual disease and with complete pathological response to treatment) and PD-L1 expression (evaluating in both cases with expression only in the lymphoplasmocytic infiltrate, and with expression in histiocytes), using the McNemar's paired chi-squared test.

Four scenarios were possible in relation to sTIL counts: (1) Increased, when sTIL counts were low/negative before and moderate/intense after NACT; (2) Positive stable, when sTILs were moderate/intense before and after NACT; (3) Negative stable, when sTILs were low/negative before and after NACT, and (4) Decreased, when sTILs were moderate/intense before NACT and turned out to be low/negative after NACT. Likewise, four scenarios were possible in relation to PD-L1 expression: (1) Increased, when PD-L1 was negative before and positive after NACT; (2) Positive stable, when PD-L1 was positive before and after NACT; (3) Negative stable, when PD-L1 was negative before and after NACT, and (4) Decreased, when PD-L1 was positive before NACT and turned out to be negative after NACT. Then, we fit a logistic regression model for the association of these four scenarios (using “Increased” as reference) with pathological response. Finally, we produced Box plots for pre- and post-NACT PD-L1 expression according to sTILs rates, including the PD-L1 expression with or without histiocytes and sTIL counts with or without pCR. T-tests were used to compare the mean values of PD-L1 expression according to sTILs status.

### Ethics statement

The study follows the recommendations of the Guiding Medical Doctors in Biomedical Research Involving Human Subjects, the Declaration of Helsinki (Declaration of Helsinki III, 1997) and Resolution 441/2012 of the National Health Council.

## Results

### Clinicopathological findings

Fifty-six patients were under 50 years of age, 107 were caucasian, and 67 were in menopause. Initial biopsy specimens showed predominance of histological non-special type (102 patients, 80.3%); Nottingham grade was 1 or 2 in 62 cases and 3 in 65; vascular invasion was present in 22 cases (16.9%). Ninety-one (71.6%) patients were positive for HR and 51 (40.2%) were positive for HER2. The minority presented Ki67 counts lower than 30% (21 patients, 16.5%) (Tables 1, 2).

**Table 1:** Correlation between the overall PD-L1 expression and high sTIL counts in the diagnostic core needle biopsies and clinical parameters.

Clinical parameters		Total N = 127*	PD-L1( +) overall N = 75 (59.0%)	OR (95%CI, *p*)^#^	sTILs (high count) N = 55 (43.3%)	OR (95%CI, *p*)^#^
Age (years)	< 50	56 (44.0%)	33 (44.0%)	Ref	24 (43.6%)	Ref
	≤ 50	71 (56.0%)	42 (56.0%)	0.99 (0.45–2,15, * p* = 1)	31 (56.4%)	0.96 (0.44–2.08, * p* = 1)
Ethnicity	Cauc	107 (84.3%)	63 (84.0%)	Ref	47 (85.4%)	Ref
	Others	20 (11.0%)	12 (16.0%)	0.95 (0.31–2.78 * p* = 1)	8 (14.6%)	1.17 (0.40–3.59, * p* = 0.80)
Menopause	No	60 (47.2%)	36 (48.0%)	Ref	27 (49.1%)	Ref
	Yes	67 (52.8%)	39 (52.0%)	1.07 (0.49–2.32, * p* = 0.86)	28 (50.9%)	1.13 (0.53–2.44, * p* = 0.72)
Pregnancy**	Yes	110 (86.5%)	64 (86.5%)	Ref	48 (87.3%)	Ref
	No	16 (13.5%)	10 (13.5%)	1.19 (0.36–4.30, * p* = 0.79)	7 (12.7%)	1.0 (0.29–3.28, * p* = 1)
Lactation***	Yes	97 (76.3%)	56 (87.5%)	Ref	41 (85.4%)	Ref
	No	13 (10.2%)	8 (12.5%)	1.17 (0.31–4.89, * p* = 1)	7 (14.6%)	1.58 (0.42–6.18, * p* = 0.55)
Hormone replacement	No	114 (89.8%)	67 (89.3%)	Ref	49 (89.1%)	Ref
	Yes	13 (10.2%)	8 (10.7%)	0.89 (0.21–3.31, * p* = 1)	6 (10.9%)	0.88 (0.23–3.38, * p* = 1)
Family history of breast and ovarian cancer	No	92 (72.4%)	52 (69.3%)	Ref	38 (69.6%)	Ref
	Yes	35 (27.6%)	23 (30.7%)	0.68 (0.27–1.62, * p* = 0.42)	17 (30.4%)	0.75 (0.31–1.76, * p* = 0,55)
Smoking	No	101 (79.5%)	61 (81.3%)	Ref	44 (80.0%)	Ref
	Yes	26 (20.5%)	14 (18.7%)	1.30 (0.49–3.39, * p* = 0.65)	11 (20.0%)	1.05 (0.40–2.80, * p* = 1)

*Cauc* Caucasian.

*Three women had bilateral breast carcinoma, sTILs: stromal tumor infiltrating lymphocytes; Ref: reference value; missing information in one case; lactation was considered exclusively for patients who had a previous pregnancy.

^#^*p value* calculation using Fisher’s exact test.

**Table 2:** Correlation between overall PD-L1 expression and high sTIL counts on diagnostic core needle biopsies and tumor parameters.

Tumor parameters		Total N = 127*	PD-L1( +) overall N = 75 (59.0%)	OR (95%CI, *p*)^#^	sTILs (high) N = 55 (43.3%)	OR (95%CI, *p*)^#^
Clinical tumor stage (Ref.^27^)	I/II	83 (65.3%)	9 (12.0%)	Ref	36 (65.5%)	Ref
	III/IV	44 (34.7%)	14 (18.7%)	0.86 (0.37–1.93, * p* = 0.84)	19 (34.5%)	1.00 (0.45–2.26, * p* = 1)
Axillary lymph node	Negative	58 (45.7%)	37 (49.3%)	Ref	27 (49.1%)	Ref
	Positive	69 (54.3%)	38 (50.7%)	1.43(0.66–3.13, * p* = 0.36)	28 (50.9%)	1.2 (0.59–2.73, * p* = 0.59)
Histological classification	Non special	102 (80.3%)	62 (82.7%)	Ref	47 (85.7%)	Ref
	Special	25 (19.7%)	13 (17.3%)	0.70 (0.26–1.86, * p* = 0.49)	8 (14.5%)	0.55 (0.18–1.50, * p* = 0.26)
Vascular invasion	No	105 (82.7%)	62 (82.7%)	Ref	46 (85.5%)	Ref
	Yes	22 (17.3%)	13 (17.3%)	0.99 (0.34–2.78, * p* = 1)	9 (16.1%)	1.12 (0.40–3.26, * p* = 1)
Grade Nottingham	1–2	62 (48.8%)	24 (32%)	Ref	18 (32.7%)	Ref
	3	65 (51.2%)	51 (68%)	**0.17 (0.07**–**0.40, *p* < 0.01)**	37 (67.3%)	**0.31 (0.13**–**0.68, *p* < 0.01)**
Molecular subtype	Luminal HER2 −	52 (40.9%)	24 (32.0%)	Ref	21 (38.2%)	Ref
	Luminal HER2+	39 (30.7%)	24 (32.0%)	0.54 (0.23–1,25, * p* = 0.14)	14 (25.5%)	1.21 (0.51–2.85, * p* = 0.66)
	Non-luminal HER2+	12 (9.5%)	6 (8.0%)	0.86 (0.24–3.01, * p* = 0.81)	5 (9.1%)	0.95 (0.27–3.39, * p* = 0.93)
	Triple negative	24 (18.9%)	21 (28.0%)	**0.12 (0.03**–**0.46, *p* < 0.01)**	15 (27.3%)	0.41 (0.15–1.1, * p* = 0.07)
Ki67	< 30%	21 (16.5%)	11 (14.7%)	Ref	7 (12.7%)	Ref
	≥ 30%	106 (83.5%)	64 (85.3%)	0.72 (0.25–2.08, * p* = 0.62)	48 (87.3%)	0.60 (0.19–1.76, * p* = 0.34)

Values in bold indicate data of statistical significance.

*Three women had bilateral breast carcinoma; sTILs: stromal tumor infiltrating lymphocytes; Ref: reference value. In one case immunohistochemistry and FISH for HER2 was inconclusive. In ten cases HER2 was not expressed through immunohistochemistry and or FISH, in percutaneous needle core biopsies (pre-treatment specimens), and was positive in the surgical specimens after NACT (post-treatment specimens). The variables with significant association with PD-L1 in univariate analysis were selected to enter a multivariate regression model; after multivariate adjustment, triple negative remained significantly associated with PD-L1 expression (*p* < 0.01; OR = 0.12, 95%CI 0.03 to 0.46).

^#^*p value* calculation using Fisher’s exact test and logistic regression.

HER2 immunohistochemical evaluation resulted in 71 negative cases (scores 0/1 +); 54 equivocal (score 2 +) and two positive (score 3 +) cases. From the 54 equivocal cases, FISH analysis resulted with gene amplification in 38 cases; 15 cases did not present gene amplification. In one case, FISH remained inconclusive (Table 2).

Complete pathological response was achieved in 25 tumors (19.7%), while in 102 there was residual disease (Table 3).

**Table 3:** Correlation between pretreatment tumor parameters and pathological response post neoadjuvant chemotherapy.

Tumor parameters		Total N = 127*	Pathological response		OR (95%CI)	*p value*^*#*^
			RCB-I to III N = 102 (80.3%)	pCR N = 25 (19.7%)		
Clinical tumor stage (Ref.^27^)	I/II	83 (65.4%)	67 (65.7%)	16 (64.0%)	Ref	Ref
	III/IV	44 (34.6%)	35 (34.2%)	9 (36.0%)	1.07 (0.37–2.90)	0.92
Axillary lymph node	Negative	58 (46.9%)	49 (48.0%)	9 (36.0%)	Ref	
	Positive	69 (53.1%)	53 (52.0%)	16 (64.0%)	1.63 (0.61–4.61)	0.37
Histological classification	Non special	102 (80.3%)	84 (82.4%)	18 (72.0%)	Ref	
	Special	25 (19.7%)	18 (17.6%)	7 (28.0%)	0.55 (0.18–1.80)	0.26
Vascular invasion	No	105 (82.7%)	84 (82.4%)	21 (84.0%)	Ref	
	Yes	22 (17.3%)	18 (17.6%)	4 (16.0%)	0.88 (0.19–3.11)	1.0
Grade Nottingham	1 + 2	62 (48.8%)	56 (54.9%)	6 (24.0%)	Ref	
	3	65 (51.2%)	46 (45.1%)	19 (76.0%)	**3.81 (1.32**–**12.6)**	** < 0.01**
Molecular subtype	Luminal HER2 −	58 (42.3%)	49 (48.0%)	3 (12.0%)	Ref	
	Luminal HER2+	28 (30.0%)	33 (32.4%)	6 (24.0%)	2.97 (0.69–12.72)	0.35
	Non-luminal HER2+	13 (9.2%)	4 (3.9%)	8 (32.0%)	**32.67 (6.13**–**174.08)**	** < 0.01**
	Triple negative	28 (18.5%)	16 (15.7%)	8 (32.0%)	**8.17 (1.93**–**34.54)**	** < 0.01**
Ki67	≤ 30%	21 (16.5%)	18 (17.6%)	3 (12.0%)	Ref	
	≥ 30%	106 (83.5%)	84 (82.4%)	22 (88.0%)	1.56 (0.40–9.03)	0.76
sTILs	Absent + mild	72 (56.7%)	62 (60.8%)	10 (40.0%)	Ref	
	Moderate + intense	55 (43.3%)	40 (39.2%)	15 (60.0%)	0.43 (0.15–1.14)	0.07
PD-L1 overall/core needle biopsy	Negative	52 (40.9%)	47 (46.1%)	5 (20.0%)	Ref	
	Positive	75 (59.1%)	55 (53.9%)	20 (80.0%)	**0.29 (0.08**–**0.89)**	**0.02**

Values in bold indicate data of statistical significance.

*Three women had bilateral breast carcinoma; pCR: pathological complete response; RCB: residual cancer burden; sTILs: stromal tumor infiltrating lymphocytes; Ref: reference value; In one case immunohistochemistry and FISH for HER2 was inconclusive. In ten cases HER2 was not expressed through immunohistochemistry and or FISH, in percutaneous needle core biopsies (pre-treatment specimens), and was positive in the surgical specimens after NACT (post-treatment specimens). Of the 75 positive cases for PD-L1, 35 expressed as much in the epithelium as in the stroma, 5 expressed only in the epithelium and 35 only in the stroma. We next fit a regression model including all variables with *p* < 0.10 (Grade—Nottingham, molecular subtype, sTILS and PD-L1); after adjustment, high histological grade, non-luminal HER2+ and triple negative carcinomas remained significantly associated with pCR: OR = 3.50, 95%CI = 1.28 to 10.7; *p* = 0.01; OR = 1.82, 95%CI = 4.41 to 90.7; *p* < 0.01 and OR = 5.0, 95%CI = 1.43 to 17.47; *p* = 0.01.

#*p* value calculation using Fisher´s exact test and logistic regression.

**Figure 1:**
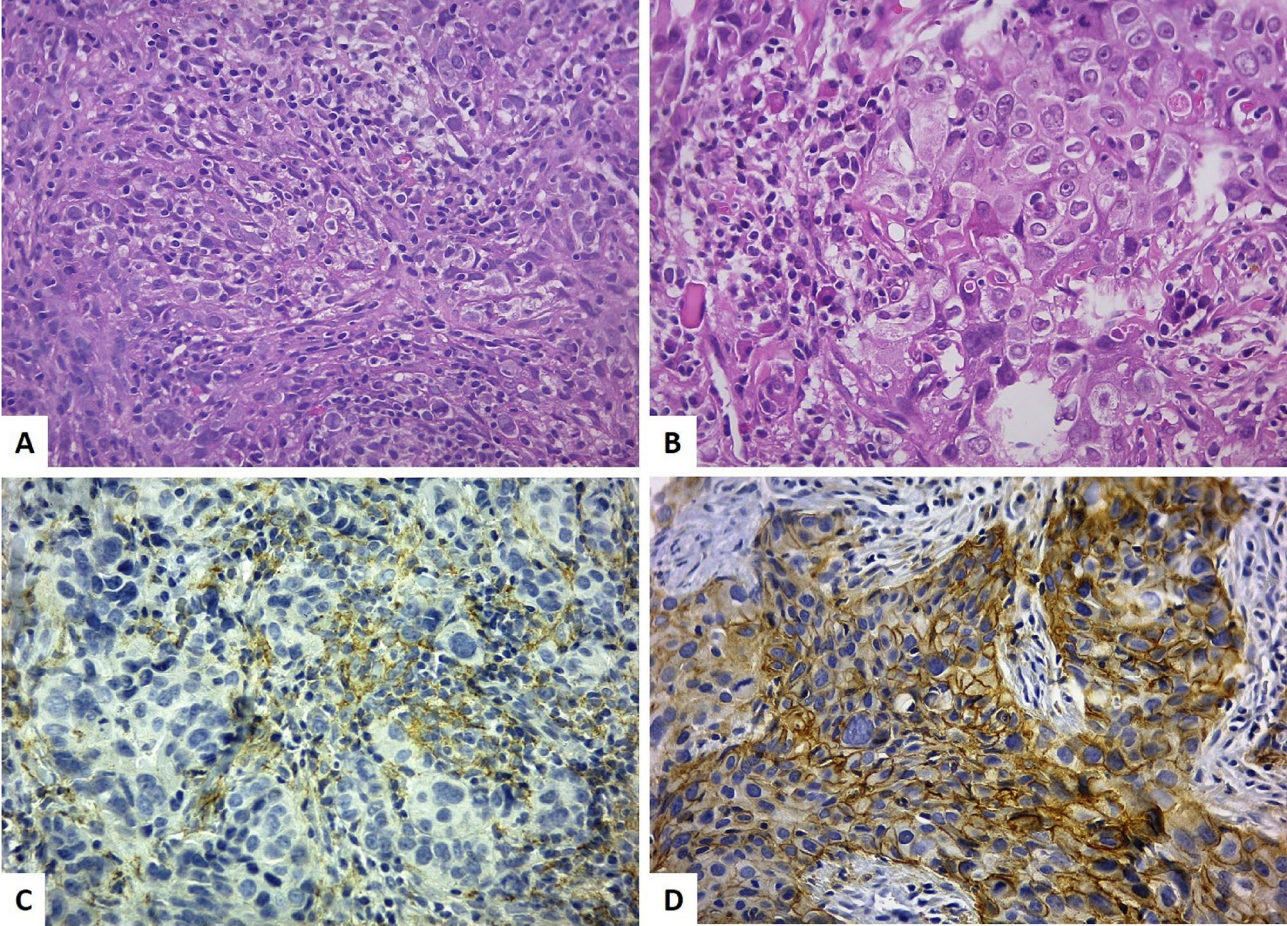
(**A**) triple negative breast carcinoma: scattered tumor infiltrating lymphocytes (core biopsy, H&E, original magnification 400 ×). (**B**) triple negative breast carcinoma: aggregates of tumor infiltrating lymphocytes (core biopsy, H&E, original magnification 400 ×). (**C**) luminal-like, HER2 negative breast carcinoma: PD-L1 positive tumor infiltrating lymphocytes (core biopsy, immunohistochemistry, original magnification 400 ×). (**D**) luminal-like, HER2 positive breast carcinoma: PD-L1 positive tumor cells (core biopsy, immunohistochemistry, original magnification 400 ×).

Among the 127 core needle biopsies collected before NACT, 75 (59.0%) expressed PD-L1 either in epithelial or stromal cells. High sTIL counts were present in 55 tumors. None of the clinical characteristics was related to both parameters (Fig. 1, Table 1).

Agreement between the two observers for the evaluation of PD-L1 status in pre-treatment biopsy was reached in 63 positive cases, and in 37 negative cases, resulting in an overall agreement (Cohen’s Kappa) of 0.54. For post-NACT specimens, agreement for positive PD-L1 status was reached in 65 cases, and in 36 negative cases, resulting in an overall agreement (Cohen´s Kappa) of 0.55. Discordant cases were mostly represented by those in which the degree of positivity was borderline.

Patients were followed up for at least 26 months, up to 56 months: 94 are alive, disease free; 11 are alive with disease and 22 died of disease. Survival analyses according to PD-L1 expression and sTIL counts in pretreatment core biopsy did not reach significant results (data not shown).

### Statistical analyses

In univariate analysis, we observed correlation between higher histological grade (Nottingham) with PD-L1 expression and high sTIL counts. The variables with significant association with PD-L1 in univariate analysis were selected to enter a multivariate regression model; after multivariate adjustment, triple negative BC remained significantly associated with PD-L1 (Table 2).

Twenty-five tumors (19.7%) of the entire cohort evolved with pCR and 102 (80.3%), with residual disease after NACT. In univariate analysis, high histological grade, and PD-L1 overall expression in core needle specimens were associated with complete response rate. After multivariate adjustment, non-luminal-HER2+ and triple negative cases were significantly associated with pCR (Table 3).

Stromal TIL counts in the core needle biopsy did not show significant change in relation to the post-NACT sTIL counts in the surgical specimen; this was valid for both, women with or without residual disease (McNemar test for paired sample *p* = 0.17 and *p* = 0.20; Table 4).

**Table 4:** Correlation of sTIL counts in paired cases, pre (core needle biopsy) and post neoadjuvant chemotherapy.

Core needle biopsy sTILs	Total (n = 122*)	Post NACT sTILs (including women with RCB and pCR)		*p value*^*#*^
		Low count n = 81 (67.7%)	High count n = 41 (32.3%)	
Low count	**68 (56.7%)**	49 (60.5%)	19 (46.3%)	0.17
High count	**54 (43.3%)**	32 (39.5%)	22 (53.7%)	

Core needle biopsy sTILs	Total (n = 100**)	Post NACT sTILs (including only women with RCB)		*p value*^*#*^
		Low count N = 62 (62.0%)	High count N = 38 (38.0%)	
Low count	**61 (61%)**	41 (67.2%)	20 (46.2%)	0.20
High count	**39 (39%)**	21 (53.8%)	18 (32.8%)	

Values in bold indicate data of statistical significance.

*Five women with RCB had no tissue post-NACT for sTILs evaluation.

**Additional twenty-two women were excluded because of pCR and three with RCBI. sTILs: stromal tumor infiltrating lymphocytes; NACT: neoadjuvant chemotherapy.

^#^*p value* calculation using McNemar's paired chi-squared.

Table 5 shows that in patients with residual tumor, 64% remained with low sTIL counts or decreased sTIL numbers and 36% remained with high sTIL counts or increased. Although not statistically significant, there was a trend to decrease in sTIL counts among patients with pCR (*p* = 0.07).

**Table 5:** Comparisons of sTIL counts in core needle biopsy and post-NACT surgical specimen according to residual cancer burden (RCB).

Comparisons of sTILs count in core needle biopsy and post-NACT	Total N = 122*	Pathological response		OR (95%CI, *p*)^#^
		RCB-I to III 97 (79.5%)	pCR 25 (20.5%)	
Increased	**32 (25.2%)**	17 (17.5%)	2 (8.0%)	Ref
Stable positive	**28 (22.0%)**	18 (18.5%)	4 (16.0%)	1.89 (0.31–11.68, * p* = 0.49)
Stable negative	**40 (31.5%)**	41 (42.3%)	8 (32.0%)	1.66 (0.32–8.83, * p* = 0.54)
Decreased	**27 (21.3%)**	21 (21.7%)	11 (44.0%)	4.45 (0.87–22.88, * p* = 0.07)

Values in bold indicate data of statistical significance.

*Five women with RCB had no tissue post-NACT for sTILs evaluation; Ref: reference value. pCR: pathological complete response; RCB: residual cancer burden; sTILs: stromal tumor infiltrating lymphocytes; NACT: neoadjuvant chemotherapy. *#p value* calculation using Pearson’s Chi-squared test.

Comparing PD- L1 status between core needle biopsy and post-NACT surgical specimens, expression status was significantly maintained: positive cases remained mostly positive and negative cases remained mostly negative. This was valid for both evaluations, considering the expression in histiocytes or disregarding these cells (McNemar test for paired sample, *p* < 0.01; Table 6).

**Table 6:** Correlation of PD-L1 expression in paired cases, pre (core needle biopsy) and post neoadjuvant chemotherapy.

Core needle biopsy PD-L1 overall	Total (n = 121*)	Post NACT PD-L1 overall with histiocytes		*p value*^*#*^
		Negative 45 (37.2%)	Positive 76 (62.8%)	
Negative	**50 (41.3%)**	30 (66.7%)	20 (23.3%)	< **0.01**
Positive	**71 (58.7%)**	15 (33.3%)	56 (73.7%)	

Core needle biopsy PD-L1 overall	Total (n = 106*)	Post NACT PD-L1 overall without histiocytes		*p value*^*#*^
		Negative 42 (39.6%)	Positive 64 (60.4%)	
Negative	**44 (41.5%)**	28 (66.7%)	16 (25.0%)	< **0.01**
Positive	**62 (58.5%)**	14 (33.3%)	48 (75.0%)	

Values in bold indicate data of statistical significance.

*Six women (two with pCR and four with RCB) had no tissue post-NACT for immunohistochemical evaluation; NACT: neoadjuvant chemotherapy; **#***p value* calculation using McNemar's paired chi-squared.

**Figure 2:**
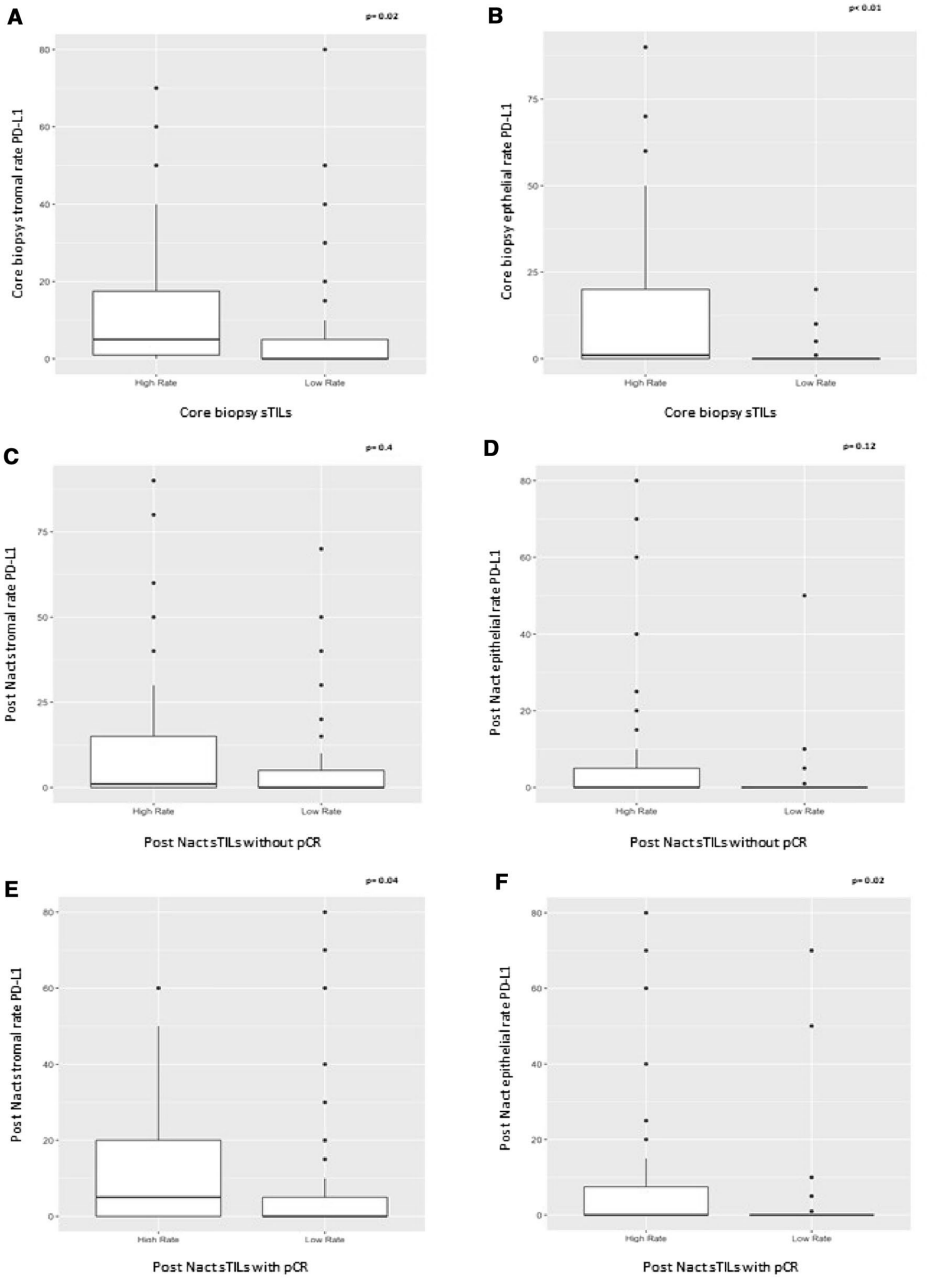
In the y-axis, PD-L1 expression is shown as the linear percentage of stained epithelial or stromal cells. In the x-axis, sTIL counts are indicated. (**A**, **C**, **E**) correspond to the values in stromal cells. (**B**, **D**, **F**) correspond to epithelial values. In core needle biopsies (pre-treatment) PD-L1 expression is correlated with sTILs, for both epithelial and stromal compartments (**A**, **B**, *p* = 0.02 and *p* < 0.01, respectively). The same happened in post-NACT specimens when all cases (with residual disease and pCR) were included (Figures E and F, *p* = 0.04 and *p* = 0.02, respectively). However, when only cases with residual disease are considered, this relation was not present (**C**, **D**, *p* = 0.40 and *p* = 0.12, respectively).

There was a significant association between stromal and epithelial PD-L1 expression with sTIL counts in pre-treatment specimens (Fig. 2A: *p* = 0.02; Fig. 2B: *p* < 0.01). In the post-NACT scenario this association also existed when all cases, with or without pCR, were included (Fig. 2E: *p* = 0.04; Fig. 2F: *p* = 0.02). No association between the expression of stromal and epithelial PD-L1 with sTIL counts was observed in the post-NACT cenario, when pCR cases were excluded (Fig. 2C: *p* = 0.40; Fig. 2D: *p* = 0.12).

When expression of PD-L1 is considered only on epithelial cells and when it is evaluated in epithelial + stromal components of core needle biopsies, it is associated with pCR (*p* = 0.02 and *p* = 0.03, respectively; Table 7).

**Table 7:** Correlation between PD-L1 expression in stroma, and in epithelial cells, in specimens pre and post-neoadjuvant chemotherapy according to pathological response.

PD-L1 protein expression		Total (n = 127)	Pathological response		*p value*^*#*^
			RCB-I to III N = 102 (80.3%)	pCR N = 25 (19.7%)	
Core biopsy—stromal cells	Positive	70 (55.1%)	52 (51.0%)	18 (72.0%)	0.09
	Negative	57 (44.9%)	50 (49.0%)	7 (28.0%)	
Core biopsy—epithelial cells	Positive	40 (31.5%)	27 (26.5%)	13 (52.0%)	**0.02**
	Negative	87 (68.5%)	75 (73.5%)	12 (48.0%)	
Core biopsy—overall	Positive	75 (59.1%)	55 (53.9%)	20 (80.0%)	**0.03**
	Negative	52 (40.9%)	47 (46.6%)	5 (20.0%)	
Post-NACT—stromal cells*	Positive	57 (44.9%)	45 (45.9%)	12 (52.2%)	0.75
	Negative	64 (53.6%)	53 (54.1%)	11 (47.8%)	
Post-NACT—epithelial cells^$^	Positive	20 (50.4%)	20 (20.6%)	–	NA
	Negative	76 (79.2%)	76 (79.2%)	–	
Post-NACT—overall	Positive	76 (59.9%)	58 (59.2%)	18 (78.3%)	0.14
	Negative	45 (35.4%)	40 (40.8%)	5 (21.7%)	

Values in bold indicate data of statistical significance.

*pCR* Pathological complete response, *RCB* Residual cancer burden, *NACT* Neoadjuvant chemotherapy.

*Six women (two with pCR and four with RCB) had no tissue post-NACT for immunohistochemical evaluation.

^$^In thirty-one cases (25 with pCR and 6 with RCB-I), PD-L1 expression in epithelial cells could not be evaluated.NA: not applicable.

^#^*p value* calculation using Fisher’s exact test.

Table 8 shows that there was no significant change of PD-L1 expression on core needle biopsy versus post-NACT surgical specimens, comparing women with residual cancer or pCR. This is valid either considering PD-L1 expression on histiocytes in the total score, or disregarding them.

**Table 8:** Comparisons of PD-L1 expression in core needle biopsy and post-NACT surgical specimen according to residual cancer burden (RCB).

Comparisons of PD-L1 expression in core needle biopsy and post-NACT	Total N = 121*	Pathological response with histiocytes		OR (95%CI, *p*)^#^
		RCB-I to III 98 (81.0%)	pCR 23 (19.0%)	
Increased	**16 (12.9%)**	16 (16.3%)	4 (17.4%)	Ref
Stable positive	**49 (39.5%)**	42 (42.9%)	14 (60.9%)	1.33 (0.38–4.66, * p* = 0.65)
Stable negative	**37 (29.8%)**	29 (29.6%)	1 (4.3%)	0.14 (0.01–1.34, * p* = 0.08)
Decreased	**22 (17.7%)**	11 (11.2%)	4 (17.4%)	1.45 (0.3–7.09, * p* = 0.84)

Comparisons of PD-L1 expression in core needle biopsy and post-NACT	Total N = 106*	Pathological response without histiocytes		OR (95%CI, *p*)^#^
		RCB-I to III 89 (84.0%)	pCR 17 (16.0%)	
Increased	**16 (15.1%)**	13 (14.6%)	3 (17.7%)	Ref
Stable positive	**48 (45.3%)**	39 (43.9%)	9 (52.9%)	1.0 (0.23–4.26, * p* = 0.10)
Stable negative	**28 (26.4%)**	27 (30.3%)	1 (5.9%)	0.16 (0.02–1.7, * p* = 0.26)
Decreased	**14 (13.2%)**	10 (11.2%)	4 (23.5%)	1.73 (0.31–9.57, * p* = 0.52)

*Six women (two with pCR and four with RCB) had no tissue post-NACT for immunohistochemical evaluation. Additional 15 cases were excluded because cellularity was constituted only by histiocytes. Ref: reference value; pCR: pathological complete response; RCB: residual cancer burden; sTILs: tumor infiltrating lymphocytes; NACT: neoadjuvant chemotherapy.

^#^*p value* calculation using Pearson’s Chi-squared test.

## Discussion

In the present study PD-L1 expression evaluated in initial biopsies correlated with pathological complete response after NACT. Significance was found either when both neoplastic and stromal cells or when just epithelial cells were considered. Similar results were reached in most studies enrolled in a systematic review by Miglietta et al.^40^. Only rare studies associated PD-L1 positive expression with the presence of residual disease after NACT^41^. According to the review by Stovgaard et al.^42^, all six studies addressed for PD-L1 expression in the NACT setting found significant association between pre-treatment PD-L1 expression and higher pCR. In this review, one of the studies showed association of PD-L1 expression in tumor cells and pCR but did not establish this marker as predictive for complete response in individual subtypes of breast carcinoma^43^. Another one found association between PD-L1 expression and pCR only in univariate, but not in multivariate analysis^44^.

Wimberly et al.^16^ favored a predictive value of PD-L1 expression for pCR applying a particular method, that is, quantitative immunofluorescence evaluation by image analysis and signal amplification with thyramide. As the values were continuous, the cut point to consider PD-L1 positive was statistically set. Cerbelli et al.^45^ showed association of PD-L1 expression in triple negative breast cancers with pCR, when cut off was set at ≥ 25% positive tumor cells. This controversy shows that studies on PD-L1 expression in breast cancer patients submitted to NACT are still needed, with special emphasis on methodological standardization of immunohistochemical evaluation and correlation with other interfering clinicopathological features. In this respect, the analyses performed in the present study, associating multiple clinical and pathological parameters with PD-L1 expression, were not homogeneously addressed in previous investigations. According to the systematic review from Stovgaard et al.^42^, PD-L1 expression in breast carcinomas variated between 0 to 83% in all different intrinsic subtypes, and most studies included in the review presented values around 50%, a figure similar to that evidenced in the present study (59%).

Comparison of IHC evaluation of PD-L1 in cancer has been problematic, as different studies use diverse anti-PD-L1 antibodies or platforms. To circumvent this issue there are currently three diagnostics automated IHC assays approved by the Food and Drug Administration (FDA), one using the SP142 clone, the other, the 22C3 clone, and the third, the SP263 clone. Not only the anti-PD-L1 antibody and the platform used are different, but also the scoring systems, with three variations: scoring on tumor cells, on stromal cells and a combination of both, tumor and stromal cells^46^. The platform using the SP142 clone is scored by ICa (percentage of tumor area covered by PD-L1 positive immune cells)^47^, and the platform using the 22C3 clone, by CPS (positive tumor or immune cells as percentage of all tumor cells), without possibility of interchange between both scoring systems^48^. For breast cancer, the ICa score has been suggested, but in a recent evaluation of the three scoring systems, Guo et al.^38^ advocated the cutoff 1% using the combined score, in tumor and immune cells.

For our study, we performed manual IHC detection of PD-L1 using the ZR3 rabbit monoclonal antibody. The ZR3 clone has been indicated as one of the biosimilar diagnostic antibodies used in clinical practice (Sorokin et al.^32^; Gonzales-Ericsson et al.^49^). Scoring was performed by a consensus of two pathologists assessing tumor epithelial cells and stromal immune cells in the tumor microenvironment by visual analysis. The scoring method used in this study for PD-L1 expression is somewhat similar to that of the SP142 assay, using a 1% cutoff, but herein this cutoff was applied to staining of both epithelial and stromal cells in combination^38,48^.

The predominant association of PD-L1 expression with non-luminal breast carcinomas (27 out of 36, or 75%) and high histological grade (51 out of 65, or 78%) found in our patients is in accordance with previous reports^16,40,42,43^. PD-L1 expression is particularly frequent in triple negative breast carcinomas, a subtype that most benefits from immunotherapy. Interestingly, among our cases of hormone positive carcinomas PD-L1 was expressed in about half of the cases (48 out of 94, or 52%). This figure is higher than the frequency reported earlier, around 20%^42^. This figure suggests that immunotherapy might also be appropriate for luminal and PD-L1 positive breast cancers, a proposition which warrants further studies.

Among pathological parameters, complete pathological response (20% of the cases) was associated with grade 3 and absent expression of hormonal receptors, results already reported before^45^. sTIL counts on initial diagnostic biopsy did not influence pathological response, in opposition with previous reports^16,18,50,51^. This discrepancy could be attributed to the different proportion of molecular subtypes of breast carcinoma in our study, or to factors related to patient’s biological characteristics. Comparing the initial biopsy with post-NACT specimens in patients with both residual tumor and complete pathological response, we observed a considerable number of cases with pCR which presented a reduction of sTILs after neoadjuvant chemotherapy. This difference, however, did not reach statistical significance (*p* = 0.07). In a report on 59 paired samples, a significant reduction in sTILs was seen in post-chemotherapy specimens in comparison with baseline samples; PD-L1 expression remained similar in the paired samples^18^. In this study the greatest decrease in sTILs was associated with pCR. In more recent studies, reduction in sTILs was also detected in post-NACT samples^50,51^. This decrease could reflect the removal of antigenic stimuli exerted by neoplastic cells, which would be followed by reduction of lymphocyte infiltrates in patients with pCR. An inverse relation, that is, an increase in sTILs in post-NACT surgical specimens, was observed by others^52,53^, who interpreted their observation as an attractant effect on lymphocytes exerted by chemotherapy. In regard to the maintenance of PD-L1 expression in residual carcinomas, it is possible that such patients could also benefit from inclusion of immune checkpoint inhibitors to conventional therapy^18^.

Our study presents some limitations, as the relatively small number of the different immunohistochemically defined subtypes of breast carcinoma; the evaluation of more patients with hormone receptor expression, HER2 positive and triple negative would allow the analysis of the clinical impact of PD-L1 expression and sTIL counts within each group. Second, the evaluation of sTILs can be made using markers for different lymphoid subtypes, allowing the determination of which could exert impact on clinical outcomes. In the present report, we used exclusively H&E, according to the guidelines of the International Immuno-Oncology Biomarker Working Group^27,28^. Finally, long term follow up could allow the evaluation of the influence of such parameters in disease free and overall survivals. However, the patients were enrolled prospectively and had a standardized treatment and evaluation.

## Conclusion

The present study supports the role of PD-L1 immunohistochemical expression evaluated in initial biopsies in predicting pCR after NACT. This is valid when only epithelial cells are considered and when both epithelial and stromal cells are included. The value of sTILs in predicting pCR was not confirmed by our data. However, we could support the notion that, in general, post-NACT specimens present decreased numbers of sTILs in patients with pCR. PD-L1 associated with higher histological grade (Nottingham) at diagnosis, with non-luminal-HER2+ and triple negative BC, and with sTIL counts. Besides, our data also support the association of pathological complete response rate with high histological grade, non-luminal-HER2+ and triple negative subtypes of BC. Of note, the higher number of patients with HR + subtype in our study might indicate a potential use of immunotherapy in this group of women. Finally, the methods used herein are applicable in most routine pathology laboratories, supported by consensus studies on scoring of sTILs and PD-L1 expression.
